# Impact of Hepatitis B Virus Genetic Variation, Integration, and Lymphotropism in Antiviral Treatment and Oncogenesis

**DOI:** 10.3390/microorganisms8101470

**Published:** 2020-09-24

**Authors:** Keith C.K. Lau, Kelly W. Burak, Carla S. Coffin

**Affiliations:** 1 Department of Microbiology, Immunology and Infectious Diseases, Cumming School of Medicine, University of Calgary, Calgary, AB T2N 1N4, Canada; kcklau@ucalgary.ca; 2 Calgary Liver Unit, Division of Gastroenterology and Hepatology, Department of Medicine, Cumming School of Medicine, University of Calgary, Calgary, AB T2N 1N4, Canada; kwburak@ucalgary.ca

**Keywords:** HBV, genetic variation, integration, treatment, oncogenesis, HCC, lymphotropism

## Abstract

Chronic Hepatitis B Virus (HBV) infection poses a significant global health burden. Although, effective treatment and vaccinations against HBV are available, challenges still exist, particularly in the development of curative therapies. The dynamic nature and unique features of HBV such as viral variants, integration of HBV DNA into host chromosomes, and extrahepatic reservoirs are considerations towards understanding the virus biology and developing improved anti-HBV treatments. In this review, we highlight the importance of these viral characteristics in the context of treatment and oncogenesis. Viral genotype and genetic variants can serve as important predictive factors for therapeutic response and outcomes in addition to oncogenic risk. HBV integration, particularly in coding genes, is implicated in the development of hepatocellular carcinoma. Furthermore, we will discuss emerging research that has identified various HBV nucleic acids and infection markers within extrahepatic sites (lymphoid cells). Intriguingly, the presence of hepatocellular carcinoma (HCC)-associated HBV variants and viral integration within the lymphoid cells may contribute towards the development of extrahepatic malignancies. Improved understanding of these HBV characteristics will enhance the development of a cure for chronic HBV infection.

## 1. Introduction

The Hepatitis B Virus (HBV) is a significant global viral pathogen. An estimated two billion individuals worldwide have been exposed to the virus with approximately 257 million living with chronic HBV infection (CHB) [1]. HBV is responsible for over 800,000 deaths a year primarily due to the induction of hepatocellular carcinoma (HCC), cirrhosis, and acute hepatitis [2]. The introduction of the HBV vaccine consisting of recombinant HBV surface antigen (HBsAg) has effectively reduced the spread of HBV, particularly in young children [1]. However, HBV remains endemic in many areas of the Southeast Asia region and Africa, which have an estimated prevalence of 6.2% and 6.1%, respectively [1,3]. Within Canada, there are an estimated 260,000 chronically infected individuals (0.76% estimated prevalence), with the majority of those with CHB are new Canadians from endemic areas [3,4]. Within the Calgary Health Zone in Alberta, Canada, our prior research study reported a total of 1214 individuals (0.10% of population within Calgary zone) who tested positive for HBsAg within a single calendar year (2014) [4]. The majority of these cases are likely newly diagnosed CHB carriers, many of which lacked appropriate monitoring and management of their disease. CHB represents a significant disease burden worldwide, and will require substantial investment in prevention, research, and patient care to achieve the World Health Organization (WHO) goals for elimination of viral hepatitis by 2030.

The HBV is transmitted both horizontally and vertically into new human hosts. Horizontal transmission may occur through exposure to materials, such as blood or semen containing infectious HBV particles. However, horizontal transmission into adult immunocompetent hosts most frequently develops into a self-limiting acute infection with clearance of HBsAg and development of HBsAg antibodies (anti-HBs). CHB is defined by positive HBsAg in serum for greater than 6 months duration. The vast majority of CHB carriers are infected by transmission of the virus by mother-to-child transmission during childbirth or close contact with blood and body fluids of other family members in early childhood (i.e., <5 years of age) [5]. In contrast, only 5% or less of adolescents and adults who are exposed to HBV eventually progress to CHB infection [6]. Individuals persistently infected with HBV are at risk of development of severe liver complications including cirrhosis and primary liver cancer (HCC). 

In this review, we will briefly discuss the natural history of CHB and the two main categories of therapeutics for CHB management (interferon and nucleos/tide analogues). It is noteworthy that persistent HBV infection has unique viral aspects, which affect antiviral treatment choices as well as oncogenic risk. These viral features include HBV genotype/genetic variations, integration of HBV DNA into host chromosomes, and recent data suggesting role of viral lymphotropism. In addition, HBV genetic variants, integration, and lymphotropism will be discussed as a consideration in the development of new antiviral therapeutics.

### 1.1. Natural History of Chronic HBV Infection

**Figure 1:**
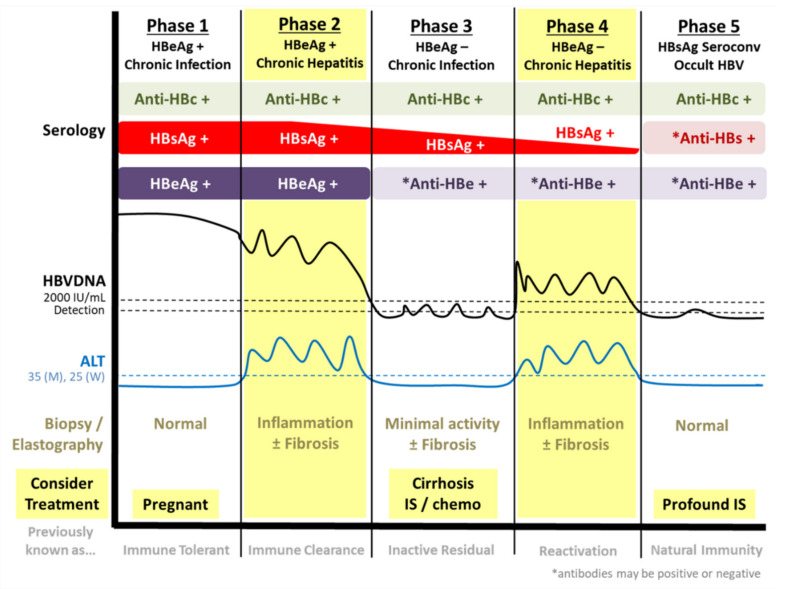
Phases of chronic hepatitis B infection (CHB) natural history. CHB is differentiated into HBeAg positive (phases 1–2) and negative phases (3–5) as well as phases of chronic hepatitis (phases 2 and 4). Chronic hepatitis phases are characterized with ALT flares, fluctuating Hepatitis B Virus (HBV) DNA levels, as well as liver damage and inflammation. Generally, anti-viral therapy is recommended during chronic hepatitis phases to reduce liver damage and suppress viral replication. However, treatment may also be beneficial for pregnant, cirrhotic, and/or immunosuppressed individuals. ALT = alanine aminotransferase; HBc = HBV core; HBsAg = HBV surface antigen; HBeAg = HBV e antigen; IS = immunosuppression.

The natural history of CHB infection is complex and has been divided into distinct phases [7,8,9]. An important viral marker utilized in the classification of CHB phases is presence or absence of viral hepatitis B “e” antigen (HBeAg). The secreted non-structural HBeAg is a non-essential secreted protein with regards to the viral replicative life cycle and has significant sequence homology to the viral HBV core (capsid) protein [7,10]. Indeed, HBeAg is an immunomodulator that has been shown to inhibit both innate and adaptive immunity through a variety of mechanisms such as interfering with interferon expression and induction of T-cell exhaustion [7,11]. The first phase is defined as a high replicative and low inflammatory “HBeAg positive infection” (Figure 1). Typically, CHB carriers in this phase are characterized with extremely high viral load (i.e., HBV DNA levels) and positivity for HBeAg [9]. Despite, the high levels of viral protein and DNA, overt liver inflammation and damage are minimal. CHB carriers may progress into the second stage, the “HBeAg positive hepatitis” phase, with hepatic flares in which liver inflammation, characterized by elevated levels of alanine aminotransferase (ALT), and damage (fibrosis) may occur [7,9].

Transition to the third stage occurs with the loss of viral HBeAg and the presence of antibodies targeting HBeAg (anti-HBe) [9]. During this phase called “HBeAg-negative infection”, individuals show lower HBV viremia and ALT with less liver inflammation. However, some patients may develop “HBeAg-negative chronic hepatitis” with higher levels of viral replication and hepatic or ALT flares [7,9]. These generally occur with the presence of HBV precore or basal core promoter (BCP) mutations that disrupt HBeAg production, but maintains or enhances viral replication [8,9,12]. BCP mutations present within the core promoter alter transcription from the viral covalently closed circular (ccc)DNA, which serves as the highly persistent template and replicative intermediate of HBV. As a consequence of BCP mutations, precore RNA, utilized to produce HBeAg, is reduced in favor of an increased pre-genomic (pg)RNA production that is required for the synthesis of new viral progeny [12]. The final phase is the HBsAg negative phase, which may occur either spontaneously or with treatment, and may be associated with the development of anti-HBs antibodies (seroconversion). This is associated with improved prognostic outcomes including reduced risks of cirrhosis and HCC [9,13]. This has been defined by the scientific community as a “functional cure” indicating robust host immune control and/or transcriptionally silent HBV cccDNA. However, only a small proportion of CHB carriers achieve a functional cure, despite many years of oral antiviral therapy with nucleos/tide analogues (NAs). A complete virological or sterilizing cure (i.e., physical clearance of HBV cccDNA and removal of integrated HBV) is not possible with currently approved therapies.

### 1.2. Current Treatment of Chronic HBV Infection

Currently approved first-line antiviral therapies for CHB infection include pegylated-interferon alpha (PEG-IFNα) or potent NAs such as tenofovir disoproxil fumarate (TDF), tenofovir alafenamide (TAF), and entecavir (ETV). HBV is a weak inducer of innate immunity (type I IFNs), thereby evading efficient immune detection and elimination of the virus [14]. Thus, the mechanisms underlying PEG-IFN targeting of HBV primarily involve harnessing the host innate immune system to target viral replication and replicative intermediates through multi-faceted approaches [15]. Indeed, studies have demonstrated the capability of IFN in inducing expression and activation of innate anti-viral interferon stimulated genes/proteins, which serves to effectively inhibit HBV replication [16,17,18,19,20,21]. Activation of interferon stimulated genes (ISGs) including, but not limited to, the apolipoprotein B mRNA editing enzyme (APOBEC)3 family, MX2, ISG20, and the TRIM family have been described with innate anti-HBV effects [16,17,18,19,20,21]. Furthermore, studies have reported the capability of IFNα therapy to inhibit viral replication and suppress HBV gene expression through epigenetic modulation of the HBV cccDNA [22,23]. PEG-IFNα treatment can be associated with higher rates of HBsAg clearance/loss (i.e., negative HBsAg serology by approved commercial ELISA assays) and, therefore, achievement of a functional cure. However, these rates seem to be genotype specific with improved treatment responses in HBV genotype A [8,24,25].

NAs inhibit the viral reverse transcriptase (RT) enzyme, an essential component in the viral life cycle, thereby attenuating production of new HBV particles. However, NAs do not directly target the HBV cccDNA and only a small minority of patients on therapy eventually develop HBsAg clearance (HBsAg negative/loss), which serves as a marker of viral suppression or functional cure [7,26]. Therefore, NAs are typically life-long treatments for CHB patients. Despite these disadvantages, NAs induces substantial reduction of HBV viral load with minimum adverse side-effects, which effectively prevents and delays the progression of CHB to cirrhosis or HCC in most patients [27,28]. Treatment for CHB is recommended primarily during phases of chronic hepatitis in which liver inflammation and fibrosis occurs alongside ALT flares (phases 2 and 4; Figure 1) [29,30]. However, certain CHB carriers will benefit from treatment during pregnancy (to prevent mother to child transmission), or if cirrhotic or immunosuppressed. In particular, numerous studies have demonstrated the effectiveness and safety of NA therapies, such as TDF, in reducing the risk of HBV immunoprophylaxis failures and mother-to-child transmission with lowering of maternal HBV viremia during pregnancy [31,32,33,34,35,36,37].

## 2. Genetic Variations within HBV

### 2.1. HBV Genotypes

**Figure 2:**
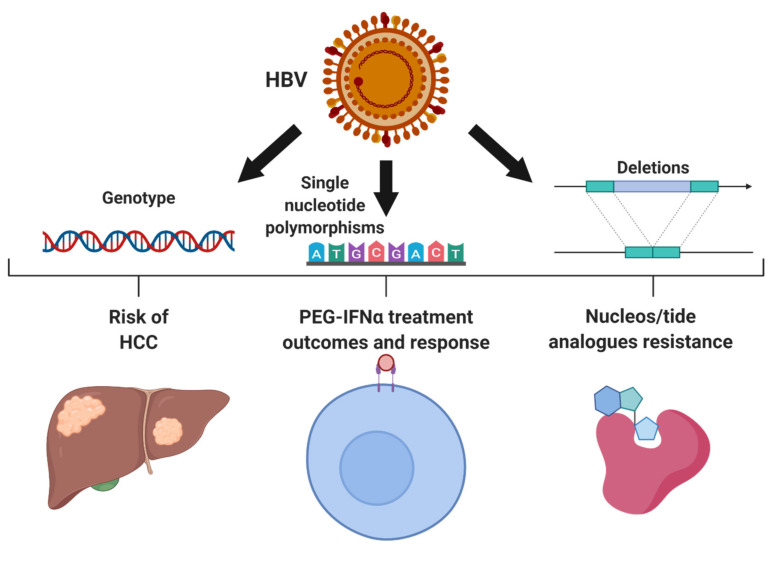
Impacts of Hepatitis B Virus (HBV) genetic variants including genotype, single nucleotide polymorphisms, and deletions of genetic regions. Certain genotypes of HBV are recognized to have higher risks of hepatocellular carcinoma (HCC) and better outcomes to anti-HBV therapeutics such as pegylated-interferon alpha (PEG-IFNα). Similarly, viral genetic polymorphisms particularly in the HBV X/basal core promoter/precore region and the surface region influence HCC risks, PEG-IFNα therapy and viral resistance to nucleos/tide analogues (NA). Figure constructed using Biorender.com.

CHB has a complex natural history, mediated by a dynamic interplay between the host immune response, susceptible host cells, and the virus. The outcome of HBV infections, response to treatment, and risk of HCC development, has been linked to viral heterogeneity. HBV genetic variants within the HBV preS1 or X/basal core promotor/pre-core (X/BCP/PC) regions are associated with treatment failure and varying risk of HCC. In addition, deletion of viral domains such as the HBV preS region may also contribute towards risks of HCC or recurrence of HCC, such as the recent reports by Teng et al. [38] HBV is currently classified into 10 different genotypes, labeled A to J, which are defined as >7.5% genetic divergence within the full genome sequence of the virus [39,40]. Furthermore, HBV is separated into a number of subgenotypes defined as 4 to 7.5% genetic divergence [41,42]. Traditionally, over 30 different HBV subgenotypes have been identified and classified [41]. However, recent studies utilizing powerful sequencing data and phylogenetic analysis have suggested a careful revision of the number and standardization of subgenotypes [42,43]. HBV genotypes are typically geographically distributed. For example, genotype B and C are endemic in the Southeast Asia, including China and Japan. Countries that experience larger influxes of immigration from a variety of regions worldwide, such as Canada, demonstrate a more diverse array of genotypes [44,45]. Different genotypes have been observed to impact treatment response as well as HCC risk, age of onset, and prognosis (Figure 2). CHB carriers with HBV genotype A show better treatment outcomes with PEG-IFNα use including HBeAg, HBsAg seroconversion, and reduced viremia [24,25,46,47,48]. In addition, comparisons between genotype B and C have revealed better outcomes to PEG-IFNα in CHB carriers with predominantly genotype B infection [48,49]. Furthermore, individuals infected predominantly with HBV genotype C or genotype D have a higher risk of developing cirrhosis and HCC in comparison to genotype B or A, respectively [39,50,51]. Other studies have demonstrated that early onset non-cirrhotic HCC is more common in HBV genotype B patients whereas genotype C is associated with later onset cirrhotic HCC [50,52,53].

### 2.2. Resistance to Anti-Viral Treatment

Due to the error-prone method of viral replication via the HBV polymerase, the HBV population within a host exists as quasi-species. Moreover, studies evaluating lymphoid cells (peripheral blood mononuclear cells (PBMCs)) in CHB carriers have reported different genotypes exist within the plasma and PBMCs [54,55]. These genotype discrepancies likely arose due to different evolutionary and environmental pressures amongst the reservoirs including antiviral treatment exposure, targeting by the immune system, or genetic drift [54,55,56]. Thus, the viral populations in these separate cellular reservoirs diverged genetically despite originating from a common source. It is important to note that CHB carriers harboring certain viral genetic variations have been demonstrated to experience different outcomes to anti-viral treatment. Specific mutations within the precore (position 1896) and BCP region (positions 1762 and 1764) of HBV are associated with reduced responses to PEG-IFNα therapy [46,57,58]. CHB carriers containing either one of more of these mutations were less likely to experience HBeAg and HBsAg clearance as well as reduction in HBV DNA in comparison to individuals harboring wild-type virus [57,58].

Mutations in HBV have also been associated with resistance against NA therapy, especially first-generation NA, such as lamivudine (Figure 2). Most of these mutations are present within the HBV polymerase, which encodes for the reverse transcriptase capability of HBV. These mutations have been well-described in previously published review articles [8,12,59,60]. Second generation oral NA therapies, such as ETV, TDF, and TAF are advantageous for a significantly higher barrier to resistance and viral breakthrough. Anti-HBV resistance to ETV requires a minimum of three different mutations with the HBV polymerase and occurs in ~1–2% of treatment naïve individuals [59]. Long-term studies have revealed continual viral suppression with TDF despite treatment for up to 10 years [61,62,63,64]. Registration studies of CHB carriers on long-term TDF treatment have reported no identifiable mutations associated with resistance after 8 and 10 years of continuous therapy [62,64]. TAF, the more recently approved formulation of tenofovir, also appears to show a high barrier to the development of viral resistance. A recent randomized trial comparing TAF vs. TDF in 1298 CHB carriers demonstrated comparable rates of virological breakthrough with no identified HBV mutations associated with treatment resistance after 96 weeks of treatment [65]. However, two recent reports have identified TDF resistance mutations within the HBV genome. Both studies report a combination of four mutations (rtL180M/T184L/M204V/A200V or rtS106C/H126Y/D134E/L269I) are required for the development of resistance [66,67]. However, these quadruple mutations were reported in only one and two CHB carriers in the respective studies and the frequency of these genetic combinations within the CHB population is likely rare. Additional research is warranted to better characterize and validate these mutations. Nonetheless, the widespread use of long-term TDF and TAF therapies outside of controlled trial studies, may lead to further issues with antiviral resistant HBV genetic variants.

### 2.3. HBV Single Nucleotide Polymorphisms Associated with HCC

In addition to influencing treatment response and outcomes, genetic variations within HBV are linked to HCC (Figure 2). Indeed, mutations within the preS1 and preS2 regions of the virus have been reported and shown to induce hepatocarcinogenesis [68,69,70]. Mutations of these regions result in impaired secretion of HBV virions and HBs subviral particles leading to an accumulation of unfolded viral proteins within the cellular ER [71,72]. It is important to note that some of the mutations may arise due to selective pressure with the use of antiviral therapies. Most notably, due to the overlapping nature of the HBV genome, the rtA181T mutation, which can confer NA therapy resistance, results the well-characterized sW172* mutation associated with truncated surface proteins [59]. The preS mutations can contribute to the histological phenotype of type II ground-glass hepatocytes [72,73]. Consequently, hepatocytes containing HBV preS mutants exhibit ER stress response, oxidative DNA damage, increased inflammation and undergo cellular apoptosis [71,72,74]. These cellular processes facilitate the neoplastic transformation of hepatocytes and the induction of HCC.

In addition to the preS mutations, genetic variants of HBV within the X/BCP/PC, have also been associated with an increased—and in some cases decreased—risk of developing cirrhosis and HCC. Some of the oncogenic variants in this region have been observed at higher frequencies in certain genotypes, which likely is reflected in the difference in HCC risks amongst HBV genotypes. For example, the double mutations A1762T and G1764A are more frequently observed in genotype C. Many variants in this region have been identified from large epidemiological studies generally from Asian populations [75]. Most notably, the A1762T and G1764A double mutations are consistently associated with HBV induced cirrhosis and HCC [68,75,76,77,78,79,80]. In addition, T1674C/G, A1752G, T1753V, T1768A, C1773T, A1846T, G1896A, and G1899A also influence HCC risks [68,75,77,78,79,80]. Amongst these variants, only the presence of A1752G is correlated with a decreased cirrhosis and HCC risk [78].

Alterations in the HBx protein are associated with mutations within the X/BCP/PC region and can lead to HCC development. Due to the overlapping nature of the HBV genome, the mutations directly affect the genetic coding sequence of HBx. A research study by Yan and colleagues exploring the quadruple mutant of A1762T/G1764A/T1753A/T1768A showed an overall downregulation of p53 in comparison to wild-type HBV X [81]. In addition, these mutations are also capable of influencing the non-structural protein, HBeAg. The variant G1896A is well recognized for the ability of introducing a premature stop codon within the HBeAg transcript resulting in a significantly truncated protein and HBeAg-negative serology [82,83]. Other mutations in this region, including the A1762T/G1764A double mutations, also result in reduced HBeAg production and expression by altering the wild-type BCP, a component of the core promoter that encodes for precore (HBeAg) RNA [83]. Interestingly, these BCP genetic variants induces a transcriptional shift resulting in an associated decrease in precore RNA (and subsequent HBeAg expression) with a corresponding increase in viral replication through enhanced pgRNA expression. Due to this phenotype, a combination of these mutations is thought to be related to the risk of hepatic flares or high viral load levels in HBeAg-negative CHB cases (Figure 1) [7,84,85]. However, many of the X/BCP/PC mutants that have been linked to HCC risk in epidemiological studies are still poorly understood particularly with regards to the molecular biology and phenotypic consequences. The overall impact of these specific genetic variants on either the viral life cycle or cellular processes are poorly characterized.

## 3. Unique Features of HBV that Impact Treatment and Oncogenesis

### 3.1. Oncogenic Implications of HBV Integration

Integration of HBV DNA into the host genome arises with the incorporation of double stranded linear (dsl) variants of HBV (Figure 2). An estimated 10% of HBV reverse transcription results in the synthesis of a dslDNA form of the HBV genome due to a lack of translocation of the viral RNA primer to the 5′ DR2 [86]. Similar to rcDNA, the dslDNA can be either exocytosed from the cell as new infectious viral progeny or recycled back into the nucleus [86]. Once the dslDNA localizes into the nucleus either through de novo secondary infection or intracellular recycling, the dslDNA is capable of inserting into the host genome, a process primarily mediated by non-homologous end joining (NHEJ) or microhomology-mediated end joining (MMEJ) that utilizes host machinery [86,87,88,89]. These cellular mechanisms of handling double strand DNA breaks are non-specific and frequently causes deletions and insertions, which are reflected within the integrated HBV [86,90]. HBV integration is not necessary for a productive HBV life cycle and results in a dead-end infection as the integrated virus is incapable of producing viral progeny [86]. It has been estimated that HBV integration occurs at a rate of 1 in 1000 hepatocytes within an infected liver [91]. Integration of HBV likely occurs shortly if not immediately after infection as suggested by studies in both the clinical and in vitro settings. HBV integration has been detected from liver tissues from early stages of CHB (immune tolerant or HBeAg+ chronic infection, phase 1 in Figure 1) [92,93]. In addition, in vitro studies have shown that HBV integration in immortalized hepatocyte cell lines can occur even within hours after entry into a new host cell [91,94].

Integration of HBV is thought to occur at random sites and has been detected in a wide variety of genes, but preferential sites for HBV integration include host genomic regions prone to dsDNA breaks or fragile sites largely due to the use of NHEJ and MMEJ mechanism [89]. Furthermore, recent studies have observed increased detection of HBV integration within coding regions, open chromatin areas, and regions with higher gene expression [95,96]. It is hypothesized that HBV integration is insufficient by itself to induce cancer, but instead facilitates hepatocarcinogenesis [97,98]. Integrations associated with oncogenic implications would likely provide survival advantages, which are selected for during clonal expansion of HCC progenitor cells. Thus, recurrent integration sites have been characterized from HCC tissues, many of which occur within genes that play a role in cellular processes implicated in oncogenesis. For example, one of the more frequently reported integration site is the human telomerase reverse transcriptase (hTERT) gene [52,96,99,100,101,102,103]. The hTERT is a well-known oncogene found to be one of the most frequent genetic alterations present in early developing HCC suggesting a role in development and pathogenesis of HCC [104,105]. This gene permits cellular immortalization by activating telomerase activity and is upregulated in cells integrated with HBV [52,96,100,101]. In addition to the hTERT, HBV recurrent integration events has been found within other genes implicated in oncogenesis and malignant transformation, such as the MLL4 and SERCA1 genes [96,101,103,106,107]. HBV integration may also influence the expression or production of non-coding RNA, which are increasingly reported to impact carcinogenesis of many cancers including HCC [108,109]. Indeed, Lau et al. has elegantly characterized a specific HBV integration site, which generates a novel long non-coding RNA (coined HBx-LINE1) with oncogenic implications [110].

### 3.2. Extrahepatic HBV Infection and Lymphotropism

**Figure 3:**
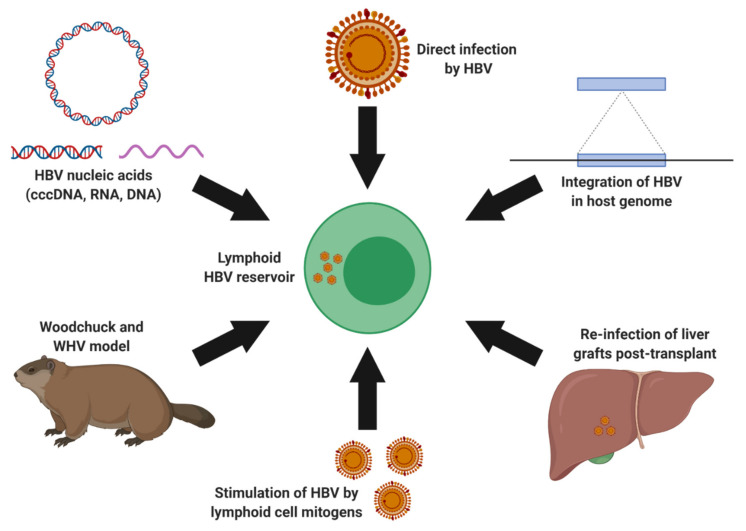
Evidence for a lymphoid HBV reservoir. The woodchuck and woodchuck hepatitis virus (WHV), a useful animal model system for hepadnaviruses, has established lymphoid infection by WHV [111,112,113,114]. Several research groups have suggested the detection of viral nucleic acids including HBV cccDNA, RNA, and DNA as well as viral integration within lymphoid cells [54,56,115,116,117,118,119,120,121,122,123,124]. Furthermore, direct infection of lymphoid cells with clinically derived HBV has been achieved in vitro [125,126]. HBV derived from the lymphoid reservoirs has been stimulated with the use of mitogens [125,127,128]. Furthermore, lymphoid derived HBV is suggested to be implicated in the re-infection of liver grafts post-liver transplant [117]. Figure created using Biorender.com.

HBV is a hepatotropic virus that infects and establishes reservoirs in hepatocytes. Studies in the woodchuck hepatitis virus (WHV) model of HBV show that WHV is also a lymphotropic virus [111,112,113,114]. Several research groups have suggested that the lymphoid system also serves as an HBV reservoir (Figure 3). Important HBV replicative intermediates and infection markers have been identified in extrahepatic lymphoid tissues including the spleen, lymphoid nodes, and lymphoid cells [54,56,115,116,117,118,119,120,121]. In fact, Lee et al., Chemin et al., and Trippler et al. have independently demonstrated that the virus is universally present in multiple immune cell subsets derived from HBV-infected individuals [122,123,124]. Studies by Yan et al. and Huang et al. reported that naïve primary PBMCs and hematopoietic stem cells, respectively, can be infected in vitro with clinically derived HBV [125,126]. Our recent work demonstrated that HBV derived from the lymphoid reservoir (PBMCs) can be stimulated with mitogens that induce an increase in multiple viral replication markers such as HBV DNA, cccDNA, and RNA both intracellularly and extracellularly [127]. Independent studies by Bouffard et al. and Yan et al. reported that comparable findings [125,128]. Limited studies of lymphoid-derived HBV have demonstrated the infectious potential of this reservoir. Brind et al. reported an important clinical implication of this extrahepatic reservoir in that PBMC-derived HBV was hypothesized as the infectious source responsible for re-infection of the liver post-transplantation [117]. The findings from these studies are particularly noteworthy as it suggests that the lymphoid cell reservoir is not only susceptible for HBV infection, but are also capable of harboring infectious HBV virions.

The presence of HBV within the lymphoid cells may also contribute towards the development of hematological neoplasms. Indeed, a number of epidemiological studies have reported associations of CHB with higher risks of non-Hodgkin lymphoma, specifically diffuse large B-cell lymphoma (DLBCL) [129,130,131,132,133,134,135,136]. Although, the mechanisms underlying this clinical association have yet to be elucidated, the direct oncogenic effects of HBV within lymphoid cells may induce malignant transformation. In support of this hypothesis, a recent study by Wang et al. not only demonstrates that B-lymphocyte cell lines are infectable by HBV, but also that DLBCL tissues contain HBV infection markers such as HBV DNA and viral HBsAg, HBcAg, as well as HBx proteins [137]. HBV integration have previously been detected within the PBMCs and hematopoietic stem cells [116,127,138]. This viral feature is well-recognized to contribute towards hepatocarcinogenesis and may also be implicated in oncogenesis of HBV infected lymphoid cells. Additional data including in-depth HBV integration studies of PBMCs and of lymphoid malignancies from CHB carriers are essential. In line with this theory, our recent work identified a total of 38 distinct HBV integration sites within the host genome, using the well-established Alu PCR technique [116,139], from PBMCs and tumor tissue derived from a CHB carrier with dendritic cell sarcoma (DCS), a lymphoproliferative disease [127]. In addition, viral proteins including HBV core and surface as well as HBV DNA, cccDNA, and RNA were detected within the DCS tumor. Next generation sequencing revealed the presence of genetic variants associated with increased HCC risk A1762T/G1764A in both the PBMC and DCS tumor [127]. Similarly, in a study of 40 HBV seronegative DLBCL patients, Sinha et al. identified occult HBV infection within 27 (67.5%) cases [140]. Furthermore, they reported the detection of HCC-associated HBV genetic variants such as X/BCP/PC and surface mutations within plasma, B-cells, and tumor tissues. The results of these recent studies highlight the potential oncological importance of HBV lymphotropism and warrant additional research to clarify the associations of HBV and hematological malignancies.

Despite, the recent advances in our understanding of HBV lymphotropism, numerous outstanding questions remain warranting additional research. Namely, the process of viral entry and the corresponding cellular receptor utilized for lymphoid cell infection remains elusive. Likely the HBV surface proteins mediate the interaction with a currently unknown host receptor. An intriguing possibility are the B-cell antigen receptors, which are specific for HBV surface proteins might serve as the mechanisms of lymphoid cell entry for HBV. Alternatively, non-specific receptors present on phagocytes may serve as a method of permitting HBV entry through the processes of phagocytosis, micropinocytosis, or endocytosis [141]. These mechanisms of viral entry has been reported in other chronic human lymphotropic viruses including hepatitis C virus, herpesviruses, and human immunodeficiency virus [141]. Furthermore, host hepatic factors generally contribute towards the enhancement of viral transcription and replication [142]. Certainly, analogous factors might exist within lymphocytes, which have yet to be identified. An intriguing theory within HBV lymphotropism could involve the infection of progenitor or hematopoietic stem cells, which leads to the subsequent dissemination of HBV fragments, nucleic acids, and particles into differentiated cells. Indeed, the prior study by Shi et al. demonstrated the possible infectivity of hematopoietic stem cells with HBV [138]. This could also explain the distribution of HBV amongst the different immune cell subtypes, including natural killer cells, monocytes, B-lymphocytes, and T-lymphocytes as previously reported [122,123,124].

Utilization of the woodchuck model and WHV could also serve to contribute towards a better understanding of hepadnaviridae lymphotropism including mechanism of viral entry, host cell restriction factors, and host factors beneficial towards viral persistence or infection. However, it is important to note that many aspects of WHV biology remains unclear including the entry mechanism into woodchuck hepatocytes. Another feature of this animal model, which is important to consider is the perceived increased lymphotropic nature of WHV in comparison to HBV. Potentially there exists certain host or viral factors, which are more permissible for WHV replication within the lymphoid cells of woodchucks. In line with this possibility, a prior study by Jin et al. have demonstrated that the WHV surface proteins, and more specifically the preS1 domain, was more efficient in interacting with woodchuck lymphoid cells in comparison to hepatocytes [143]. This increased affinity for lymphoid cells might account for the selective infection of lymphoid cells at low levels of WHV as demonstrated by Michalak et al. [111]. Identification of additional factors implicated in the permissibility of WHV lymphotropism may contribute towards an understanding of the HBV lymphoid reservoirs present within CHB carriers.

## 4. Considerations for Novel HBV Therapeutics

### 4.1. HBV Genetic Variation as Predictor Factors

A wide variety of novel therapeutics are under development for CHB infection, hoping to achieve a functional (HBsAg loss) and/or sterilizing cure (clearance of HBV cccDNA). These include capsid assembly inhibitors, HBV entry inhibitors, and immunotherapies, such as checkpoint blockade inhibitors and RIG-1 agonists (see https://www.hepb.org/treatment-and-management/drug-watch/ for more) [144]. An important consideration moving forward is the role of HBV genotypes and genetic variants as predictors of treatment response and outcomes. Indeed, an enhanced understanding of viral genetic variations and their association with treatment response would be beneficial in the development of individually tailored curative therapy. Similar to the predictive value of genotype utilized to select patients more likely to respond to PEG-IFNα therapy, careful patient selection may guide the development of antivirals to benefit all persons with HBV infection, regardless of disease phase. Clinical studies with novel HBV treatment will need to consider the role of novel genetic tools and HBV biomarkers, such as high throughput sequencing and genotyping to characterize the study population and their unique viral variants.

### 4.2. Occult HBV Infection

Due to the nature of HBV integration and HBV cccDNA persistence, a sterilizing cure that eliminates all viral genomic material from infected hosts is extremely challenging. Instead, current and developing antiviral therapies focuses on achieving a “functional cure” in which viral activity is suppressed, transcriptional silencing of HBV cccDNA, with seroconversion of HBsAg (loss of HBsAg with development of anti-HBs antibodies, Phase 5 in Figure 1) [145]. Achieving HBsAg loss and a functional cure does not fully eliminate the risk factors associated with persistent HBV and can lead to occult hepatitis B infection (OBI). OBI is a condition defined primarily with HBsAg-negative serology despite the presence of replication competent HBV [146]. OBI cases may or may not have detectable HBV viremia due to frequently suppressed viral expression and replication. This suppression may arise primarily due to host immune system control of HBV (Figure 1) [147,148]. Typically, individuals with OBI are identified with positive HBV genome detection from at least two HBV genomic regions using highly sensitive molecular techniques, such as nested polymerase chain reaction (PCR) [146]. Nonetheless, detection and accurate genotyping of the HBV can be particularly challenging in OBI individuals with very low-level HBV viremia (<10 IU/mL or 50 virus copies/mL).

Despite the HBsAg-negativity and low-level viremia, OBI remains clinically significant as HBV may reactivate with immunosuppression and individuals with OBI have increased risks of malignant disease relative to HBV-negative individuals [149,150]. A prior study following 1217 Alaskan Natives an average of 19.6 years revealed no differences amongst those who cleared HBsAg (OBI) and those who remained HBsAg positive [151]. Indeed, HBV replicative intermediates can be frequently detected and found in HCC patients without overt CHB disease [152,153,154,155]. In fact, many cases of cryptogenic HCC (those with no known cause) have been linked to OBI [155,156,157]. Studies have demonstrated that oncogenic viral integration is detectable and likely poses a continual risk for malignant transformation of hepatocytes [139,157,158]. In addition to HCC and liver disease, reports have suggested that the presence of occult low-level HBV are also associated with extrahepatic and hematological malignancies including leukemia and lymphoma [159,160,161]. HBV reactivation in OBI may also be particularly problematic within patients who undergo chemotherapeutics or immunosuppressive therapies that impact host adaptive immunity. The most well-characterized cases occur in patients receiving anti-CD20 therapy (e.g., rituximab) [162,163,164]. As a consequence of the disruption of host anti-HBV immunity, the virus can reactivate potentially leading to acute liver failure and fatal outcomes. These situations emphasize the appropriate identification of OBI to prevent and monitor potential HBV reactivation.

### 4.3. Therapeutic Management of HBV Lymphoid Reservoirs

HBV lymphotropism is an intriguing consideration in the natural history of HBV infection, particularly if lymphoid cells may serve as a viral reservoir capable of producing infectious HBV. Most pre-clinical studies of HBV therapeutics involve the in vitro use of immortalized cell lines (e.g., HepG2, HepG2.2.15, Huh7, and/or HepaRG) with and/or without the HBV entry receptor, sodium taurocholate cotransporting polypeptide (NTCP), as well as in vivo murine models (e.g., uPA/SCID) with humanized liver. However, it may be of interest to assess in vitro or in vivo systems for the effect of developing therapies on hepadnaviral infection within the lymphoid reservoir. The murine HBV models with humanized liver usually have defective immune systems and cells to allow for growth of xenograft liver tissue [165] and contain non-humanized lymphoid cells, which are likely not permissible to HBV infection. Thus, the development of a CHB infection system within a lymphoid cell line or primary PBMCs would be useful as an in vitro model. For in vivo studies, the use of immunocompetent animals with lymphoid cells that are naturally infected by hepadnavirus could serve as useful pre-clinical models. As previously noted, Eastern North American woodchucks (*Marmota monax*) susceptible to WHV, is a highly valuable animal model for human HBV infection with closely related hepadnaviral infection. Numerous studies have demonstrated the lymphotropic nature of WHV, which can replicate and produce infectious virions within the woodchuck lymphatic system [111,112,113,114].

In summary, there has been rapid development of direct acting antivirals and host targeting novel HBV agents. Increased understanding and development of tools to study the complex virology of HBV and the dynamic interplay with the host immune response is needed to ultimately achieve a HBV cure. Viral genetic variations can impact therapeutic development, such as refining the selection and monitoring of potential treatment responders. The role of HBV lymphoid reservoirs as a source of liver cell re-infection, especially in OBI, and extrahepatic complications remains controversial. Moreover, even patients who achieve a functional cure (HBsAg clearance) late in life, may harbor OBI and remain at risk for HCC and overt HBV reactivation with potent immunosuppression.
